# Shellfish sanitation monitoring in La Spezia gulf: Chemometric evaluation of data from 2015 to 2021

**DOI:** 10.1016/j.heliyon.2023.e17032

**Published:** 2023-06-15

**Authors:** Erica Vaccaro, Valentina Ciccotelli, Paolo Oliveri, Roberta Battistini, Cristina Capelli, Stefano Lottici, Nunzia Melchiorre, Elena Smirnova, Marta Ferro, Erica Costa, Barbara Betti, Barbara Vivaldi, Chiara Masotti, Laura Serracca, Francesco Iacona, Mino Orlandi, Carlo Ercolini

**Affiliations:** a Istituto Zooprofilattico Sperimentale del Piemonte, Liguria e Valle d'Aosta, Turin, Italy; b Dipartimento di Farmacia (DIFAR), Università degli Studi di, Genova, Italy; c ARPA, Liguria, Italy; d Liguria Local Health Unit-ASL5, Complex Unit of Hygiene of Food and Animal Origin, La Spezia, Italy

**Keywords:** Marine biotoxins, Chemometric analysis, Principal component analysis, Shellfish sanitation monitoring, Environmental contaminants

## Abstract

Shellfish sanitary controls are very important to guarantee consumer health because bivalve molluscs (BVM) are filter-feeders so they can accumulate pathogens, environmental contaminants and biotoxins produced by some algae, causing infections and food poisoning in humans after ingestion. The purpose of this work was to analyse with chemometric methods the historical data relating to routine analyses carried out by the competent authority (Liguria Local Health Unit, National Health Service) on the BVM reared in a shellfish farm located in the Gulf of La Spezia (Italy). Chemometric analysis was aimed at identifying any correlations between the variables, as well as any seasonal trends and similarities between the stations, in order to be able to provide further material for a more accurate risk assessment and to improve the monitoring organization for example by reducing sampling stations and/or sampling frequency. The dataset used included 31 variables classified as biotoxicological, microbiological and chemical variables, measured twice a week, monthly or half yearly respectively, for a total of 6 years (from 2015 to 2021), on samples of *Mytilus galloprovincialis* coming from 7 monitoring stations. The results obtained by the application of principal component analysis have shown positive alga-biotoxin correlations, as well as seasonal trends linked to algae growth, with a greater algal biomass and their toxins during the spring months. In addition, periods characterised by low rainfall were found to affect algal development, promoting especially species such as *Dinophysis* spp. Considering the microbiological and biotoxicological variables, significant differences between the monitoring stations were not found. However, stations could be distinguished on the basis of the nature of the predominant chemical pollutants.

## 1 Introduction

Mussel cultivation (*Mytilus galloprovincialis*) in the Gulf of La Spezia dates to 1887 and constitutes an important and traditional element of the local economy. The shellfish farming activity is based on a single cooperative company: the breeding extends over an area of 583,639 m^2^ and with a production of about 25,000 quintals of product per year. The Gulf contains within it numerous anthropogenic and industrial activities, such as shipbuilding [1].

Bivalve molluscs are filter-feeding organisms: they filter plankton and other nutrients found in seawater, but also pathogens, biotoxins and environmental contaminants and, consequently, accumulate them in the tissues. Through the food chain, these harmful substances reach humans causing infections and poisoning, the symptoms and severity of which depend on the pathogen or molecule itself [[2], [3], [4]]. Both bacterial and viral microbiological agents, such as *Salmonella* spp., some Vibrios, norovirus and the virus responsible for hepatitis A (HAV), can cause infections, while environmental contaminants, such as heavy metals, polycyclic aromatic hydrocarbons (PAHs), dioxins (PCDD and PCDF) and polychlorinated biphenyls (PCBs), together with the biotoxins produced by certain algae, are responsible for food poisoning [5]. Community regulations 853/2004 [6] and 854/2004 [7] call for frequent checks along the entire supply chain, with regard to the breeding areas, which are classified according to the concentrations of *Escherichia coli*, used as an indicator of faecal contamination in Europe [8,9].

Microalgal biotoxins are secondary metabolites synthesized by some species of algae, such as dinophytes of the genus *Alexandrium*, *Gymnodinium, Dinophysis, Prorocentrum* and diatoms of the genus *Pseudo-nitzschia*, for protective and offensive purposes, therefore, to inhibit the growth of other surrounding microalgal species and the attack of predators [10]. Marine biotoxins are heterogeneous compounds, characterized by complex structures and a different degree of hydrophilicity/lipophilicity. A first classification can be made based on the molecule polarity: fat-soluble biotoxins include molecules responsible for Diarrhetic Shellfish Poisoning (DSP); instead, water-soluble biotoxins are responsible for Paralytic Shellfish Poisoning (PSP) and Amnesic Shellfish Poisoning (ASP). A joint consultation of FAO/IOC/WHO experts which met in 2004 in Oslo [11] developed a better classification of algal biotoxins based on their chemical structure: domoic acid (DA) group; okadaic acid (OA) group; azaspiracids group (AZA); brevetoxin group; group of cyclic imines; pectenotoxin group (PTX); saxitoxin group (STX); yessotoxin group (YTX).

Domoic acid group includes domoic acid and its isomers, mainly produced by algae of the genus *Pseudo-nitzschia* [12] and responsible for the Amnesic Shellfish Poisoning. These molecules are rare amino acids with neuroexcitatory activity: since their structure is similar to kainic and glutamic acid, they act as NMDA, AMPA and kainate receptors agonists [12]. This interaction results in an increase of Na^+^, Ca^2+^ and Cl^−^ incoming flows and consequently water, which cause at first cellular swelling and then death [13]. These injuries are mainly located in the CA_1_ and CA_3_ regions of the hippocampus: hence the major symptom of the intoxication which is the short-term memory loss [14].

Diarrhetic Shellfish Poisoning is caused by toxins belonging to the okadaic acid group, such as okadaic acid and its derivatives dinophysis toxins, produced by algae of the genus *Dinophysis* [15] and *Prorocentrum* [16]. Okadaic acid is a polyether derived by a C_38_ fat acid with four hydroxyl groups [17], which, together with the carboxyl group, are essential for the binding to its target [18,19]. Diarrhoea is the most common symptom of DSP (followed by nausea, vomiting and abdominal pain) and it is the result of a hyperphosphorylation of intestinal proteins, which control sodium secretion, due to the reversible inhibition of protein phosphatases 1 and 2A by the toxins [20,21].

Azaspiracids are a family of polyethers produced by algae of the genus *Azadinium* spp. and characterised by an azaspiro ring and a terminal carboxyl acid [22], hence the name of the toxins. They are responsible for the Azaspiracid Poisoning (AZP) [23]: the clinical picture is characterised by nausea, vomiting, severe diarrhoea and gastrointestinal pain, which can last for three days, but without consequences [24].

Saxitoxin and its analogues are alkaloids produced by algae of the genus A*lexandrium, Gymnodinium catenatum* and Pyrodinium bahamense [25]. They are characterised by a trialkyltetrahydropurine group and two guanidinium groups [26] which are essential for the binding with the target: these groups, positively charged at physiological level, interact with the negative charges of amino acid residues of the voltage-gated sodium channels. The result is a block of the incoming flow of sodium and consequently the propagation of action potential in excitable cells [27]. This mechanism of action makes the STX one of the most powerful neurotoxins known [28] and it is responsible, with its analogues, for Paralytic Shellfish Poisoning. The most common symptoms are nausea and vomiting, muscular weakness, respiratory distress and in severe cases also respiratory and muscular paralysis and heart failure [29,30].

Yessotoxins are a family of molecules mainly produced by algae of the genus *Protoceratium reticulatum* [31], *Lingulodinium polyedrum* [32,33] e *Gonyaulax spinifera* [34], but some of these toxins are the product of shellfish metabolism. The basic chemical structure is a polyether with 11 *trans*-fused rings [35]. YTXs target and mechanism of action is still not known, and so also symptoms it can cause, since there have never been cases of intoxication in humans. However, many studies on mice were performed in order to outline the toxicological profile: after an intraperitoneal injection of a lethal dose of YTX, mice have shown dyspnoea, restlessness, cramps and convulsions [36,37]. No symptoms were noticed after oral injection [[36], [37], [38], [39]]. However, in both cases cardiac morphological alterations were observed [36,38].

The microorganisms that can be found in live bivalve molluscs can be indigenous to the aquatic environment, such as *Vibrio* spp*.*, or, in most cases, they are pathogens deriving from the warm-blooded animal gastrointestinal system. These pathogens reach the waters through the sewage system, such as members of Enterobacteriaceae (e.g., pathogenic *E. coli*, *Salmonella*), pathogenic enterococci, *Campylobacter* and others are from aquatic environment and soil such as *Aeromonas*, *Arcobacter*, and *Pseudomonas* [40]. Among the indigenous bacterial pathogens, *Vibrio* spp. occurring naturally in brackish and marine waters, *Vibrio parahaemolyticus*, *V. vulnificus*, and *V. cholerae* are the main species involved in seafood- and seawater-borne illness worldwide. The major human pathogenic *Vibrio* species are *V. parahaemolyticus, V. vulnificus*, and *V. cholerae*. The severity of human disease caused by the different species varies considerably. *Vibrio* species are commonly associated with gastroenteritis of varying severity except *V. vulnificus* infections that can result in septicaemia with a high mortality rate [41]. Among faecal pathogens, the bacteria mainly coming from livestock animals upstream coastal areas such as non-typhoidal *Salmonella* and *Campylobacter* spp. are leading causes of bacterial gastroenteritis in many countries. Salmonellosis can be due to numerous serovars of *Salmonella enterica* subspecies *enterica,* but few of them (such as Typhimurium and its monophasic variants or Enteritidis) are cause of most human infections [42]. With over 229,000 human cases a year, campylobacteriosis is the most frequently reported food-borne illness in EU mainly due to *Campylobacter jejuni* followed by far by *C. coli*, whereas *C. lari* is implicated in a lesser extend [42]. *Campylobacter* has been frequently detected in waters at the level of catchments and in coastal areas [43,44], however, few shellfish outbreaks due to *Campylobacter* have been reported [45]. *E. coli* is a commensal of the intestinal microflora of humans and animals, however some strains are pathogenic bacteria even such that producing Shiga-toxin (STEC) or enteropathogenic *E. coli* (EPEC). These strains are able to cause pathologies that could potentially lead to haemolytic uremic syndrome. Members of *E. coli* are also responsible for extra-intestinal pathologies including urinary tract infections, meningitis or septicaemia [46]. Since the last decades, enterococci have become nosocomial pathogens of global importance where *Enterococcus faecium* and *E. faecalis* are clinically the most feared species [47]. Finally, in addition to human bacterial pathogens, enteric viruses such as norovirus (NoV) and the virus of hepatitis A (HAV) are the viruses most often associated with gastroenteritis due to consumption of shellfish worldwide. NoVs are a group of highly diverse viruses that belong to the *Caliciviridae* family, cause gastroenteritis characterized by vomiting, abdominal cramps, fever, watery diarrhoea, headaches, chills and myalgia, that normally lasts 2–3 days [48]. Hepatitis A is one of the most serious pathologies associated with the consumption of contaminated bivalves. The responsible virus belongs to the genus Hepatovirus. Infection manifests as fever, malaise, loss of appetite, diarrhoea, nausea, abdominal pain, dark urine and jaundice [49].

Another source of risk linked to the bivalve mollusc consumption is constituted by the possible presence of chemical contaminants such as heavy metals, radionuclides, polycyclic aromatic hydrocarbons, dioxins and polychlorinated biphenyls. The main heavy metals that are monitored in bivalves are mercury, cadmium and lead: these are non-essential and toxic elements, which do not perform functions in normal biochemical processes and for which the European legislation [50] set maximum allowable levels. Also silver, arsenic, chrome, copper, nickel and zinc are monitored in order to assess the quality of the water used for aquaculture: these metals, unlike the previous ones, belong to the group of essential elements, so they become toxic only at high concentrations [51]. The presence of metal traces in the seas is due both to the fact that they are natural components of rocks and sediments and to industrial activities, the disposal or incineration of waste and agricultural and zootechnical practices [52].

Dioxins and polychlorinated biphenyls are semi-volatile and thermostable substances, insoluble in water and resistant to chemical and biological degradation. Due to their lipophilic nature, once they reach the aquatic environment, they tend to adsorb to suspended particles and then, they accumulate in the tissues when ingested by marine organisms. Through the food chain, these compounds can reach humans in much higher concentrations due to the phenomenon of biomagnification [53]. Limited exposure over time to high levels of dioxins can cause impaired liver function and skin diseases, such as chloracne. On the other hand, chronic exposure to low concentrations can cause damage to the immune, endocrine and reproductive systems. In addition, dioxins can have a carcinogenic effect, as has been shown for 2,3,7,8-tetrachloro-dibenzo-p-dioxin (TCDD), classified by the IARC in Group 1 of carcinogenic substances for humans [54,55]. Polycyclic aromatic hydrocarbons (PAHs) are a large class of poorly polar organic compounds, inert and with high melting and boiling points. They derive from the incomplete combustion or pyrolysis of organic material during industrial processes; only a few of them are intentionally produced, such as naphthalene, used as an anti-moth, phenanthrene, pyrene, anthracene, fluoranthene and acenaphthene, used as intermediates in the production of plasticizers, pigments, dyes and pesticides [56]. Due to their high lipophilicity, once ingested or inhaled, they are rapidly absorbed through the gastrointestinal tract or the pulmonary epithelium and accumulate in the tissues. They undergo phase I biotransformation reactions led by enzymes of the cytochrome P450 family, which lead to the formation of epoxides and hydroxylated compounds, capable of interacting with different macromolecules, including DNA [57]. Only benzo[*a*]pyrene has been classified by IARC as a human carcinogen (Group 1); many others fall into Group 2A or 2B, probable or possible carcinogens respectively [58].

In December 2021, new community guidelines on live bivalve molluscs were approved [59], relating to phytoplankton potentially producing marine biotoxins and the classification and microbiological monitoring of production and relaying areas, whereas the community guidelines on marine biotoxins remain in preparation. The latter report the criteria for the classification of production and relaying areas and the sampling procedures for phytoplankton and bivalve molluscs. Microbiological and environmental contaminants previously described are taken into account for the classification, instead the sampling frequencies depend on the results of risk analysis carried out by the competent authority, considering the indications contained in the guidelines. Since all these analyses require a huge effort on the part of samplers and laboratories, the aim of the present work is to process the data collected from 2015 to 2021 with chemometric methods.

Several authors have studied some of these parameters both to verify correlations already demonstrated and to reveal links between different parameters, for example Sunda [60] proved that heavy metals, especially iron, have a major impact on toxin production in harmful microalgae by limiting nutrient availability and slowing down their growth, so Walha et al. [61] evaluated the relationship between heavy metal concentrations and the proliferation of potentially harmful dinoflagellates on three different matrices: tidal flat waters (TFW), sediments and urigidum in a tidal coastal area of the Gulf of Gabes (South of Tunisia). Bazzoni through a multidisciplinary approach, monitored the presence of numerous pathogenic bacteria (*E. coli*, *Salmonella* spp., and *Vibrio* spp.), viral pathogens (HAV and NoV genogroups I and II [GI and GII]), phytoplankton composition and associated algal biotoxins (PSP, DSP and other lipophilic biotoxins) in Lake Calich (north-western coast of Sardinia, Italy) to improve the knowledge on their diffusion, in order to evaluate the potential risk for public health and to implement rational conservation and management strategies of this transitional ecosystem, in support of a future request for classification of the lagoon for shellfish production [62].

The innovation of this work is the chemometric approach. Through the application of mathematical and statistical processes, chemometrics enable the extraction of the maximum content of meaningful information from larger datasets of related variables compared with those used in traditional techniques, which have specific limitations. In fact, it's proven itself to be a strong tool for analysing data in the environmental field as it can interpret both quantitative and qualitative techniques and identify any trends, not visible in other way [63,64]. Given the novelty of this approach we have tried to relate a greater number of variables and we hope to provide further material to improve the monitoring organization, for example by reducing sampling stations and/or sampling frequency.

## 2 Materials and methods

### 2.1 Sampling

**Fig. 1:**
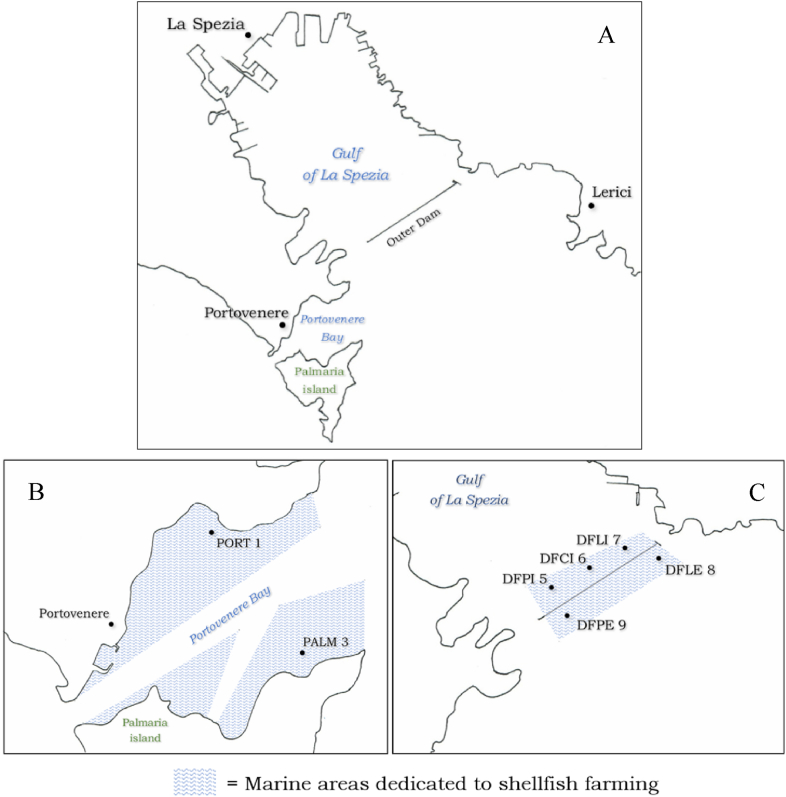
Illustration of the Gulf of La Spezia (A) and detail of the areas dedicated to shellfish farming: Portovenere Bay (B) and Outer dam (C). PORT 1, PALM 3, DFPI 5, DFCI 6, DFLI 7, DFLE 8 and DFPE 9 monitoring stations are shown according to the coordinates given in Table 1.

The areas intended for the production of bivalve molluscs (*Mytilus galloprovincialis*), shown in Fig. 1A, are divided into specific zones as outlined in Table 1 and shown in Fig. 1B and C. Seven monitoring stations have been identified, based on the sources of environmental contaminants and on the hydrodynamic and currents typical of the Gulf. Geographical coordinates are reported in Table 1.

**Table 1:** Areas for shellfish farming in the Gulf of La Spezia: they are divided into 7 monitoring stations (zone). Sampling points are identified by the geographical coordinates.

Area	Zone	Geographical coordinates
**Portovenere Bay**	PORT 1	Lat 44.057892 N Long 9.843861 E
	PALM 3	Lat 44.050794 N Long 9.850206 E
**Outer Dam**	DFPI 5	Lat 44.072528 N Long 9.858883 E
	DFCI 6	Lat 44.075725 N Long 9.865947 E
	DFLI 7	Lat 44.078431 N Long 9.871744 E
	DFLE 8	Lat 44.077233 N Long 9.880128 E
	DFPE 9	Lat 44.070111 N Long 9.862522 E

For each monitoring station, mussels were collected grouped in different points and, moreover, at three levels of depth (on the bottom, in the centre and at 50 cm from the surface), to obtain an aliquot of about 2–3 kg for each point. After collection, samples were stored at 4 °C and transferred within 4 h to the IZS and ARPAL laboratories that carried out the chemical and microbiological analysis as soon as possible. At each point, water samples were collected for phytoplankton assessment with an Apstein's net with a mesh diameter of 20 μm, by means of a vertical draw from the bottom to the surface; in addition, three further samples were made with a Niskin's bottle at three depths (surface, mid-water and bottom) to ensure a more representative evaluation of the real concentration of each algal strain possibly present. The sampling rate varied according to the season, as reported in Table 2.

**Table 2:** Frequencies of analytical controls on bivalve mussels.

Period	Microbiological quality control	Biotoxicological quality control	Viral quality control	Chemical quality control
May 1st – September 30th	Monthly	Bi-weekly	Monthly	Six-monthly^a^
October 1st – April 30th	Monthly	Monthly	Monthly	Six-monthly^a^

a Radionuclide analyses have annual frequency.

These controls can be intensified when the attention levels of the phytoplankton abundances are exceeded. These precautionary levels are set at values that have never been associated to significative biotoxins concentrations since 2000, acting as an early warning system (Table 3).

**Table 3:** Attention levels for phytoplankton in seawater: for precautionary purposes they are set at values that have never been associated to significative biotoxins concentrations.

Taxon	Attention levels (cell L^−1^)
*Alexandrium taylorii*	≥10,000
*Alexandrium* spp*.* and *Gymnodinium catenatum*	≥500
*Azadinium* spp*.*	≥10,000
*Dinophysis* spp*.*	≥500
*Gonyaulax spinifera*	≥500
*Lingulodinium polyedra*	≥500
*Ostreopsis* spp*.*	≥10,000
*Prorocentrum lima*	≥500
*Protoceratium reticulatum*	≥500
*Pseudo-nitzschia* spp*.*	≥500,000

Data collected and processed covered a period of approximately six and a half years, from January 2015 to June 2021. Variables measured on the samples are classified in: biotoxicological variables, such as PSP toxins, domoic acid, okadaic acid, yessotoxins, azaspiracids and their respective algae *Alexandrium* spp*., Pseudo-nitzschia* spp*., Dinophysis* spp*., Gonyaulax* spp*., Lingulodinium polyedrum, Protoceratium reticulatum* and *Azadinium* spp*.*; microbiological variables, such as *E. coli, Salmonella* spp*., V. parahaemolyticus, V. cholerae, V. vulnificus*, HAV and norovirus; and chemical variables, such as Hg, Cd, Pb, As, Zn, Cu, Cr, Ni, PCDD/Fs, indicator PCBs, sum of PCDD/Fs and dl-PCBs, PAHs.

Data used for chemometric elaboration are presented in graphs in the supplementary material.

### 2.2 Reagents and chemicals

Reagents used for analysis of algal biotoxins were LC-MS grade. Methanol and ammonium hydroxide (30%) were purchased from Carlo Erba (Milan, Italy), hydrochloric acid (37%) and sodium hydroxide (99%) were obtained from Sigma Aldrich (Steinheim, Germany). Trifluoroacetic acid (TFA, 99%) was purchased from Panreac AppliChem (Milan, Italy). Certified reference material for DA and DSP toxins (OA, DTX1, DTX2, YTX, HomoYTX, AZA1, AZA2, AZA3) were purchased from the National Research Council (Halifax, Canada). A customized multi-element standard solution (CPA chem**,** Bogomilovo**,** Bulgaria) was used to prepare heavy metals calibrants. As internal standard was used Rhodium standard solution 1000 mg l^−1^ **(**Merck, Darmstadt, Germany) and Yttrium standard solution 1000 mg l^−1^ (VWR Pennsylvania, USA). A multi-element standard solution N9301721 Instrument Calibration Standard 2 (PerkinElmer, Waltham, MA) was used to check calibration. High-purity deionized water (Milli-Q system, Millipore, Bedford, MA) was used for dilution of samples and standards; 60–65% ultrapure-grade HNO_3_ used for digestion was purchased from Sigma-Merck.

Standard solutions for dioxin and PCBs were purchased from Cerilliant Corporation (Texas, USA). Acetone, n-hexane, nonane and toluene pesticide grade were obtained from Lab Service Analytica (Bologna, Italy). For PAHs analysis, acetonitrile (ACN) and methyl alcohol LC-MS grade were purchased from Carlo Erba, QuEChERS 6000 mg MgSO_4_/1500 mg NaCl and Bond Elut Dispersive SPE 15 mL, Fatty samples AOAC were purchased from Agilent Technologies (Palo Alto, CA, USA), Dr Ehrenstorfer PAH mix 45 were purchased from Lab Service Analytica (Bologna, Italy), for the study, the following PAHs were selected: acenaphthylene, fluorene, phenanthrene, anthracene, pyrene, benzo[*a*]anthracene, chrysene, benzo[*b*]fluoranthene, benzo[k]fluoranthene, benzo[*a*]pyrene, indeno [1,2,3-c,d]pyrene and dibenzo[a,h]anthracene. Stock, intermediate and working standard solution PAHs were prepared in acetonitrile. Deionized water (18 MΩ) was produced by a Milli-Q system (Millipore; USA).

### 2.3 Biotoxins analysis in mussels

The preliminary treatment of the samples was common to all biotoxins analysed and included: cleaning by washing the bivalve molluscs with running water, opening the valves with a flat surface knife and collecting the pulp in a colander. Then, three portions of 2.0 ± 0.05 g, 4.0 ± 0,1 g and 5.0 ± 0,1 g of homogenised are collected in the polypropylene tubes for the analysis of DSP, DA and PSP biotoxins, respectively.

Lipophilic marine biotoxins are quantified with a LC-MS/MS method tested on a secondary validation according to “EU-RL-MB: SOP for lipophilic marine biotoxins, version 5 (EURLMB, 2015)”. HPLC Accela coupled with a TSQ Vantage (Thermo Fisher Scientific Scientific, USA) with a Chromatographic Kinetex EVO (100 × 2.1 mm 2.6 μm – Phenomenex, Torrance, CA, USA) column was employed for the separation and quantification of the lipophilic marine biotoxins. The mass acquisition was achieved in multiple reaction monitoring (MRM). Mobile phases consisted of (A) 0.05% NH_3_ aqueous solution (v/v) (B) 0.05% NH_3_ in ACN:H_2_0 (90:10 v/v) with flow at 450 μL min^−1^. The gradient elution was as follows: from 0.0 min to 1.0 min at 5% of B, from 1.0 min to 13 min at 90% of B and at the last re-equilibration at initial conditions for 5 min. The LOQs of method were 20 μg/kg for OAs and AZAs and 50 μg/kg for YTXs, with repeatability <8.5%, for all the species. Determination of domoic acid was performed with a validated method according to “AESAN EU-RL-MB Domoic acid, version 1 (EURLMB, 2008)”. The extract, was loaded into a HPLC-DAD (1100 Series, Agilent Technologies, Palo Alto, CA, USA) equipped with a Synergy 4 μm Polar RP column (250 × 4.6 mm, 4 μm – Phenomenex, Torrance, CA, USA) maintained at the temperature of 25 °C. This analysis was conducted in isocratic elution with a mobile phase composed of ACN/H_2_O (10:90 v/v) at 0.02% TFA with flow rate at 1 mL min^−1^. The LOQ of the method was 5 mg kg^−1^, repeatability and reproducibility (expressed as RSD%) were respectively <7.7% and <12.7%, with uncertainty ranging from 23% at the LOQ level to 17% at the maximum level (40 mg kg^−1^). A screening of PSP toxins was obtained by Biological Method (AOAC official method 959.08, 2000) but, from 2019, a screening based on the application of the AOAC official method 2005.06, 2006 has been applied.

### 2.4 Phytoplankton analysis in seawater

After sampling, seawater was collected in dark glass bottles with airtight cap. Lugol solution was used as a fixative. The quantification of phytoplankton was carried out with the Utermöhl method, according to UNI EN 15204:2006, which requires a first sedimentation of the phytoplankton, count using inverted microscope with 400× magnification and abundance calculation.

### 2.5 Microbiological analysis

#### 2.5.1 *Salmonella* spp.

Determination of *Salmonella* spp*.* was carried out according to UNI EN ISO 6579–1:2020. Three different culture media (non-selective pre-enrichment, selective enrichment and solid agar media) were used sequentially, interspersed with incubation periods.

#### 2.5.2 *E. coli*

*E. coli* was identified according to UNI EN ISO 16649–3:2015: the number of colony-forming units (CFU) of β-glucuronidase-positive *E. coli* was determined after incubation of the sample in a Tryptone Bile Glucuronide (TBX) medium plate.

#### 2.5.3 Isolation and identification of *Vibrio parahaemolyticus*, *Vibrio cholerae* and *Vibrio vulnificus*

Determination of *Vibrio parahaemolyticus, Vibrio cholerae* and *Vibrio vulnificus* in mussels was carried out according to ISO 21872–1:2017 [65]. Under aseptic conditions, 25 g of flesh and intervalvular water were collected, transferred to a sterile bag and added with 225 mL of Peptone Alkaline Salt Water (APWS). Samples were homogenised in a Stomacher 400 lab blender (PBI International) for 2 min and incubated for 6 h at 41.5 °C (for *V. cholerae* and *V. parahaemolyticus*), or at 37 °C (for *V. vulnificus*), for its first enrichment and cell growth. An aliquot (1 mL) was inoculated into 9 mL of APWS (second enrichment) and incubated at 41.5 °C, or at 37 °C, for 18 h. At the same time, an aliquot was streaked in Thiosulfate Citrate Bile Salts Sucrose Agar (TCBS) and CHROMAgar Vibrio. These media were then incubated at 37 ± 1 °C for 24 ± 3 h. A second passage on TCBS and CHROMAgar Vibrio was done, starting from the second enrichment broth. Blue-green colonies on TCBS and mauve colonies on CHROMAgar Vibrio were considered typical of *V. parahaemolyticus*, blue-green colonies on TCBS and green-blue to turquoise blue colonies on CHROMAgar Vibrio agar were considered typical of *V. vulnificus,* yellow colonies on TCBS and blue colonies to turquoise blue on CHROMagar Vibrio, were considered typical of *V. cholerae*. A maximum of 5 colonies typical for *V. parahaemolyticus, V. vulnificus* and *V. cholerae* per plate of selective medium were isolated on saline nutrient agar (SNA) and then analysed with biochemical tests for species confirmation.

### 2.6 Viral analysis

#### 2.6.1 Samples processing

Mussel samples were processed according to ISO 15216–2:2019 method [66]. Briefly, for each sample, hepatopancreas of 10 animals were dissected, finely chopped, and 2 g of the pool were homogenised with TissueLyser (Qiagen). Homogenates were spiked with 10 μL of process control virus Mengovirus (MV) and added with 2 mL of proteinase K solution. Samples were incubated at 37 °C, with shaking at 350 rpm for 60 min, followed by 15 min at 60 °C in a water bath. After centrifugation at 3000×*g* for 5 min, supernatant was recovered for RNA extraction and volume recorded.

#### 2.6.2 *RNA* extraction

RNA extraction was performed on 500 μL of the supernatants, with a commercial kit based on selective binding of nucleic acids to silica magnetic beads (Nuclisens® Magnetic Extraction Kit – NucliSEN easyMAG system; BioMérieux, Marcy I’Étoile, France) following manufacturer's instructions. RNA was eluted into 100 μL of elution buffer and was either immediately used for the NoV and HAV detection or stored at the temperature of **−**80 °C until analysis.

#### 2.6.3 Real time RT-PCR

Real-time RT-PCR for NoV and HAV detection was carried out on the Bio-rad CFX96TM Real-Time PCR system (Bio-rad, Hercules, CA, USA) using reagents concentrations, primers, probes, and amplification conditions as described in ISO 15216–2:2019. According to the ISO method, only values of NoV and HAV in samples with extraction efficiency ≥1% and RT-PCR inhibition ≤75% were considered valid.

### 2.7 Environmental contaminant analysis

#### 2.7.1 *Heavy* metals

Heavy metals (Ag, As, Cd, Cr, Ni, Pb, Cu, Zn) were quantified according to EPA method 3051A 2007 (microwave-assisted acid digestion, performed with ultrapure nitric acid) [67] and EPA 6020B 2014 (inductively coupled plasma-mass spectrometry analysis method) [68].

A CEM EXPLORER SP-D 24/48 was employed for sample acid digestion. Ag, As, Cd, Cr, Ni, Pb, Cu and Zn were determined by ICP-MS, PerkinElmer NexION 350X, equipped with an autosampler Elemental Scientific Inc. (ESI, USA) PrepFAST mod. SC-2DXX.

NexION 350X has a collision/reaction cell to improve signal to background measurements for many elements that suffer from spectral interference. For this study, helium gas (purity >99.9999% purity) was used as the collision cell gas for KED mode (Kinetic Energy Discrimination) and oxygen gas (purity >99.9995%) was used as the reactive cell gas, in DRC mode (Dynamic Reaction Cell).

Further details are summarised in Table 4.

**Table 4:** Parameters used in multi-elements analysis.

**Element**	**Mode**	**Detection limit [mg kg**^**−**^**^1^]**
^107^Ag	KED (S-SQ-KED)	0.05
^111^Cd	KED (S-SQ-KED)	0.001
^208^Pb	KED (S-SQ-KED)	0.01
^103^Rh (IS)	KED (S-SQ-KED)	/
^66^Zn	KED (S-SQ-KED)	1
^89^Y (IS)	KED (S-SQ-KED)	/
^60^Ni	KED (S-SQ-KED)	0.05
^63^Cu	KED (S-SQ-KED)	0.05
^89^Y (IS)	KED (S-SQ-KED)	/
^75^As |^75^As.^16^O	DRC (S-TQ-O_2_)	0.01
^52^Cr |^52^Cr.^16^O	DRC (S-TQ-O_2_)	0.03
^89^Y|^89^Y.^16^O (IS)	DRC (S-TQ-O_2_)	/

#### 2.7.2 *Dioxins* and PCBs

The seventeen 2,3,7,8-substituted CDDs/CDFs were quantified according to U.S. EPA Method 1613B 1994 “Tetra-through Octa- Chlorinated Dioxins and Furans by Isotope Dilution HRGC/HRMS”, by high resolution gas chromatography/high resolution mass spectrometry (HRGC/HRMS) [69].

The chlorinated biphenyl congeners in tissue matrices were analysed by isotope dilution and internal standard HRGC/HRMS, according to the U.S. EPA Method 1668C 2010 [70].

In accordance with the COMMISSION REGULATION (EC) No 1881/2006, results were reported as a “sum of polychlorinated dibenzo-para-dioxins (PCDDs) and polychlorinated dibenzofurans (PCDFs), expressed as World Health Organization (WHO) toxic equivalent using the WHO-toxic equivalency factors (WHO 2005 TEFs) and sum of dioxins and dioxin-like PCBs (sum of PCDDs, PCDFs and polychlorinated biphenyls (PCBs), expressed as WHO toxic equivalent using the WHO 2005 TEFs”.

Samples were previously freeze dried using the Martin-Christ freeze dryer. Extraction of PCDD/F and PCBs was performed by DIONEX ASE200 and ASE350, whereas the purification of the extracts was performed on an automatic system Miura followed by microconcentration with a CentriVap system.

In the present study, an HRGC/HRMS Thermo Scientific DFS was used, with electron-impact ionization at 45 eV and split/splitless injector in splitless mode. Mass acquisition was achieved in multiple ion detection mode (MID). The columns used were J&W DB5ms 60 m × 0.250 mm ID x 0.10 μm film for dioxin analysis, and SGE HT8 60 m × 0.250 mm ID for PCBs analysis.

#### 2.7.3 PAHs

PAHs were determined according to an internal method. A dispersive solid-phase extraction was performed, using QuEChERS methodology, followed by UPLC with fluorescence detection (UPLC Acquity Waters, Milford, MA, USA).

### 2.8 Chemometric data analysis

Chemometric analysis was carried out on a dataset composed by 31 variables measured on 798 samples of *Mytilus galloprovincialis*. Variables are divided as follows:

• Biotoxicological variables, which include PSP toxins, DA, OA, YTXs and AZAs and their causative algae *Alexandrium* spp., *Pseudo-nitzschia* spp., *Dinophysis* spp., *Gonyaulax* spp., *Lingulodinium polyedrum*, *Protoceratium reticulatum* and *Azadinium* spp.;
• Microbiological variables, which include *E. coli*, *Salmonella* spp., *V. parahaemolyticus, V. cholerae, V. vulnificus*, HAV and norovirus;
• Chemical variables, which include Hg, Cd, Pb, As, Zn, Cu, Cr, Ni, PCDD/Fs, indicator PCBs, sum of PCDD/Fs and dl-PCBs, PAHs.

Positive random values below half the limit of quantification (LOD) were assigned to values that were originally lower than this limit whereas, in the case of “positive” or “negative” results related to virus or vibrio presence/absence, a dummy 0.1 value was arbitrarily attributed to codify positive samples and 0.001 ± 0.0005 to the negative ones. Since PSP toxins were never found in these areas, but they must be always determined according to regulations, random values below one tenth of the law limit had assigned them, in order to include them in the statistical analysis, considering that they might be found in future sampling campaigns. In addition, variables that showed a highly asymmetric distribution in a first exploratory univariate analysis were normalised by applying a logarithmic transform. Autoscaling was applied to all variables as the column pre-treatment.

Statistical analysis was performed using an exploratory chemometric technique known as Principal Component Analysis (PCA) with the R-based software package CAT (Chemometric Agile Tool) [71].

## 3 Results

All the algae showed a similar trend over the years, in fact their abundance was higher during the spring and summer months. The highest abundance of *Alexandrium* spp. was observed on May 2017, when a total density of 8900 cell L^−1^ was registered in PALM. Even though this value, and two more in May 2017 (520 cell L^−1^ in PALM 3 and 640 cell L^−1^ in DFPI 5), were above the attention level, concentration of PSPs was always below the detection limit in all stations. The median value of *Pseudo-nitzschia* spp. was 420 cell L^−1^, the highest abundance was of 2,314,600 cell L^−1^ detected in June 2016 in DFPI 5. The abundance was higher than the attention level in May, June and August 2016, July 2018 and September 2020. Quantifiable concentrations of domoic acid (limit of 20 mg kg^−1^ of edible part) were observed from April to September (much more frequent in September) only for the years of 2015, 2016 and 2017; the median was 7.9 mg kg^−1^, with values ranging from 5 mg kg^−1^ to 25.8 mg kg^−1^ (maximum value in PALM 3, September 2015). When above the detection limit, it was present in all sampling stations. Abundances of *Dinophysis* spp. above the attention level were found in May and June 2016, May 2017 and January 2020, with the highest value of 81,020 cell L^−1^ detected in June 2016 in DFPI 5. OA showed the same trend in all sampling stations with values mostly below the detection limit; the highest values were 165.8 and 162.6 μg kg^−1^, found in May 2016 in the DFLI 7 and DFLE 8 points, respectively, slightly higher than the maximum content set at 160 μg of OA eq kg^−1^ of shellfish edible parts.

The highest abundances of YTX-producer dinoflagellates *Gonyaulax* spp. *L. polyedra* and *P. reticulatum* were 81,020 cell L^−1^ (May 2016, DFPI 5), 29,673 cell L^−1^ (September 2020, DFCI 6) and 1540 cell L^−1^ (June 2016, PORT 1) respectively. *Gonyaulax* spp. was recorded above attention level in May 2016, May, June and September 2017, August 2019; the abundance of *L. polyedra* when detectable was mostly above the attention level, especially between May and September; *P. reticulatum* was above the attention level in May 2016 and in May, June 2017. YTXs were often present in concentrations below the legal limit (3.75 mg of YTX eq kg^−1^ of edible part) but were more frequent than OA; the trend was different depending on the sampling point: higher concentrations, around 1.9 and 3.0 mg kg^−1^, were measured in the DFPI 5 and DFPE points in May 2015 and in August 2019.

*Azadinium* spp. has rarely been observed: only in April 2018 it has exceeded the level of attention, however azaspiracids were always below the detection level.

The median value of *E. coli* was 78 MPN/100 g, the highest value (>18,000 MPN/100 g) was found in DFPE 9 in July 2015. The results for *E. coli* were compared with the rainfall data of the days prior to sampling, to verify the presence of a connection between the microorganism concentration in the MBV and a greater supply of water in the breeding area. It was not possible to establish a direct relationship; however, the highest values of *E. coli* were found in the winter months, suggesting a seasonal trend. *Salmonella* spp*., Vibrio parahaemolyticus* and *Vibrio cholerae* and hepatitis A virus (HAV) were always absent at all sampling stations. As for norovirus, the site where it was found most frequently is DFPI 5 (55%, or 43 positivity found on 78 samples analysed); for the other sampling sites, the prevalence was in any case between 34% and 53% positivity in winter months.

As regards the chemical parameters, the analysis showed values always below the legal limits. Heavy metals recorded average values of 0.018 mg kg^−1^ for Hg (with a maximum of 0.065 mg kg^−1^ in PORT 1 in November 2018), 0.067 mg kg^−1^ for Cd (with a maximum of 0.60 mg kg^−1^ in DFLI 7 in May 2015), 0.68 mg kg^−1^ for Pb (with a maximum of 1.2 mg kg^−1^ in DFLI 7 in November 2017), 2.36 mg kg^−1^ for As (with a maximum of 4.3 mg kg^−1^ in DFLE 8 in May 2017) and 0.57 mg kg^−1^ for Cr (maximum value of 1.4 mg kg^−1^ in PORT 1 in May 2016). The values of dioxins, PCBs, PCB-DL and PAHs were always well below the maximum levels established by the EC Reg. 1881/2006.

## 4 Discussion

Our observations confirmed what extensively discussed by Zingone [72]: DSP toxins and DSP-producer algae (*Dnophysis* spp. and *Prorocentrum lima*) are more present in the northern regions of the Adriatic Sea, along the French and Spanish coasts of the Mediterranean Sea, more occasionally in the Eastern Mediterranean and Tunisian waters, causing large economic losses to aquaculture. In the last decade, however, no cases of serious poisoning have been recorded. DTX analogues, DTX-1 and DTX-2, were never detected in the sampled mussels, perhaps because they were acylated derivatives found only in the digestive system glands of contaminated shellfish and are not believed to be phytoplankton metabolites [73].

The YTX-producer dinoflagellates *Gonyaulax spinifera*, *Lingulodinium polyedra* and *Protoceratium reticulatum* are quite widespread in the Mediterranean Sea and they adapt to several conditions such as temperature, salinity, pH or nutrients [74]. The analysis of the seasonal trend in YTXs contamination in the North-central Adriatic Sea suggests that autumn-winter is the most critical period of the year [75], instead in the Gulf of Spezia we observed a two-year trend: between 2016 and 2017 there was a prevalence in the winter season, between 2018 and 2019 in the summer period and finally in 2020 and in the first half of 2021 an increase in YTXs was recorded again in the winter months; however, the observed concentrations are much lower than those of the Marche region.

*Pseudo-nitzschia* blooms are widespread, but domoic acid in shellfish rarely exceeds regulatory levels, indeed low values of DA have occasionally been found in shellfish from the Adriatic Sea, Greece and in mussel samples from mid-Tyrrhenian waters [72].

For the first time a significant PSP contamination emerged in Sicilian molluscs. The recurring blooms of *Alexandrium* spp. in spring-summer 2015–2017 and the high levels of contamination measured in mussels (maximum content of total PSP of 1508 μg STX eq kg^−1^ in May 2015 and 4131 μg STX eq kg^−1^ between April and May 2017) highlight a food safety problem [76]. In contrast, such in-depth studies have not yet been carried out in the Ligurian Sea, but monitoring program results exclude a danger to human health.

AZAs has been detected in areas such as England, Norway or Spain [74], so they are typical of very cold seas, even if, due to globalization, traces of about 7 μg AZA eq kg^−1^ were detected in the edible tissue of molluscs from the central-northern Adriatic Sea for the first time in July 2012 and 2013, showing a predominance of AZA-2 [75], while its presence has never been observed in the region object of study.

As regards the microbiological contamination, the seasonal trend of norovirus is confirmed with a prevalence in the winter season as reported by Battistini et al. [77] in a study carried out on oysters in the same hydrological basin between 2018 and 2019.

Toxic metals such as mercury, cadmium, chromium and arsenic have been found at concentrations similar to what reported by Squadrone et al. [78] while we recorded lower values for lead (0.068 mg kg^−1^ compared to 0.49 mg kg^−1^) and higher values for arsenic (2.36 mg kg^−1^ compared to 0.93 mg kg^−1^). In any case, the values were lower than the current maximum limits set by the European Community, confirming that the product can be considered absolutely safe for human consumption.

Basic statistical processing did not highlight correlations; the same data were, therefore, analysed with multivariate statistical methods.

PCA was first carried out on microbiological and biotoxicological variables together and, subsequently, on chemical variables, separately. The two lowest-order PCs were considered in both of the cases, which together explained 17.3% of the total variance of microbiological and biotoxicological data, and 48.3% of the total variance of the chemical ones.

**Fig. 2:**
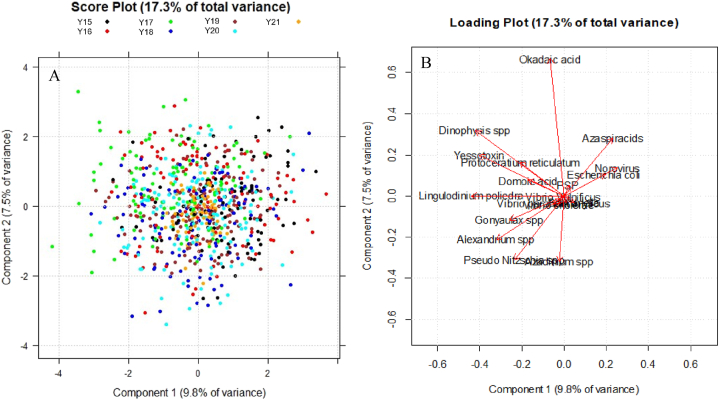
Score plot (A) and loading plot (B) in the plane of the two lowest-order PCs. Samples in the score plot are identified with different colours on the basis of the monitoring year. (For interpretation of the references to colour in this figure legend, the reader is referred to the Web version of this article.)

Concerning microbiological and biotoxicological data, in the score plot in Fig. 2A samples are identified with different colours corresponding to the sampling year. It can be noticed that data distribution is rather homogeneous, except for year 2017, which is mainly located towards negative values of PC1 and positive values of PC2 scores. Comparing this score plot with the loading plot in Fig. 2B, this shift can be associated to a higher presence of algae, such as *Dinophysis* spp. and *Protoceratium reticulatum*, but also yessotoxins.

**Fig. 3:**
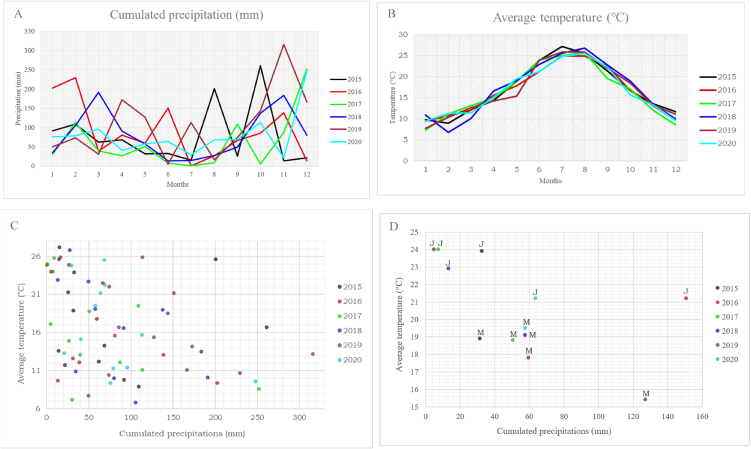
(A) Monthly cumulated precipitation patterns (in mm) from 2015 to 2020. (B) Monthly average temperature (in °C) from 2015 to 2020. (C) Average temperature patterns (in °C) in function of the cumulated precipitations (in mm) from to 2015 to 2020. (D) Average temperature patterns (in °C) in function of the cumulated precipitations (in mm) from 2015 to 2020, only for the months of May (M) and June (J).

By examining historical meteorological data recorded at the weather station of La Spezia (SPZIA) reported on the Liguria Region website [79] and related to monthly cumulated precipitations and monthly average temperatures, year 2017 appeared to be characterised by low precipitations, especially during the months of May and June (Fig. 3A and B). This is much more evident in the bivariate analysis, which considers monthly cumulated precipitations and monthly average temperatures together (Fig. 3C and D).

A similar profile occurred for year 2018: in the score plot in Fig. 2A, a partial overlap can be actually observed, in the second quadrant, between the samples from these two years.

We therefore suggest that a mild temperature, around 20 °C, and low rainfalls might promote algae proliferation, of those algae located in the second quadrant of the loading plot (*Dinophysis* spp. and *Protoceratium reticulatum*). This experimental result finds a match in the scientific literature: in an article published on 2013, Vlamis A. and Katikou P. [80] recorded *Dynophysis* spp. abundances in Greek seas from 2003 to 2008 and studied them with climatological parameters, such as air temperature, rainfall, wind direction and velocity. They found out that a higher abundance (cells L^−1^) of algae of the genus *Dinophysis* was more frequently recorded with no or low rainfall in combination with air temperature in the range of 4–20 °C.

**Fig. 4:**
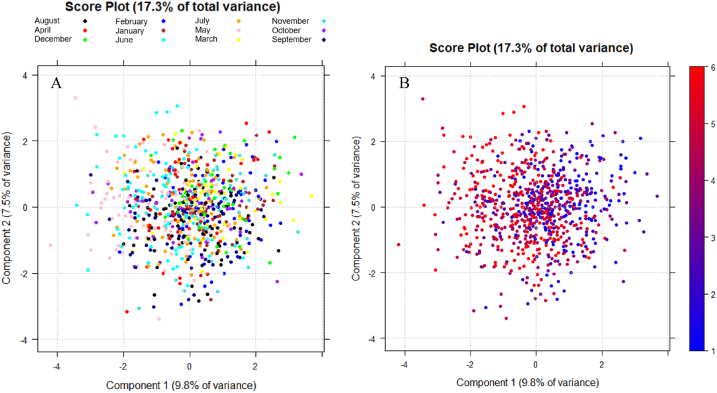
(A) Score plot in the plane of the two lowest-order PCs. Samples are shown with different colours based on the sampling month. (B) Score plot in the plane of the two lowest-order PCs. Samples are differentiated on the basis of the sampling month using a chromatic scale from blue to red; 1 = January and *February,* 2 = December and March, 3 = November and April, 4 = October and May, 5 = September and June, 6 = August and July. (For interpretation of the references to colour in this figure legend, the reader is referred to the Web version of this article.)

In the score plot in Fig. 4A, samples are differentiated with different colours based on the sampling month. Here, PC1 is related to seasonality: spring months, such as May and June, are located towards negative values, whereas colder months (from November to March) can be found towards the positive ones. The chromatic scale adopted in the score plot in Fig. 4B makes more evident the seasonal trend. If the first score plot in Fig. 4A is compared with the loading plot in Fig. 2B, it can be noticed that milder months are characterised by a higher presence of algae and their biotoxins, except for *Azadinium* spp. and its biotoxins azaspiracids, which are located towards positive values of PC1. This experimental result is in accordance with the fact that algae of the genus *Azadinium* spp*.* are typically found in colder seas [2].

**Fig. 5:**
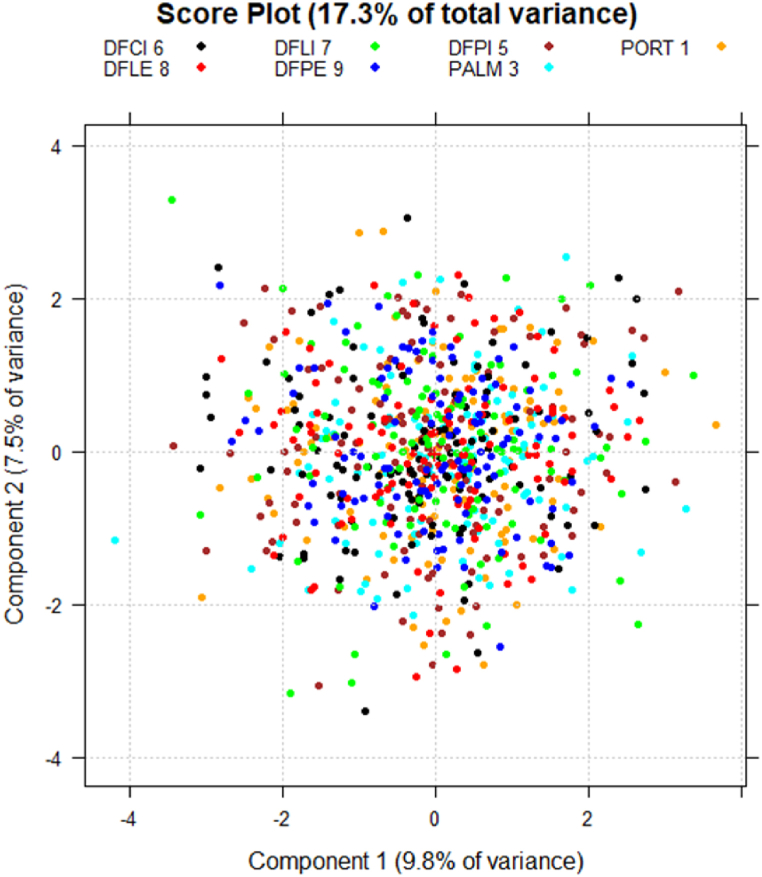
Score plot in the plane of the two lowest-order PCs. Samples are identified with different colours based on the monitoring station of origin. (For interpretation of the references to colour in this figure legend, the reader is referred to the Web version of this article.)

Considering only microbiological and biotoxicological parameters, no significative difference between the monitoring stations is detectable in the PC score space, if points are coloured according to the stations (Fig. 5).

**Fig. 6:**
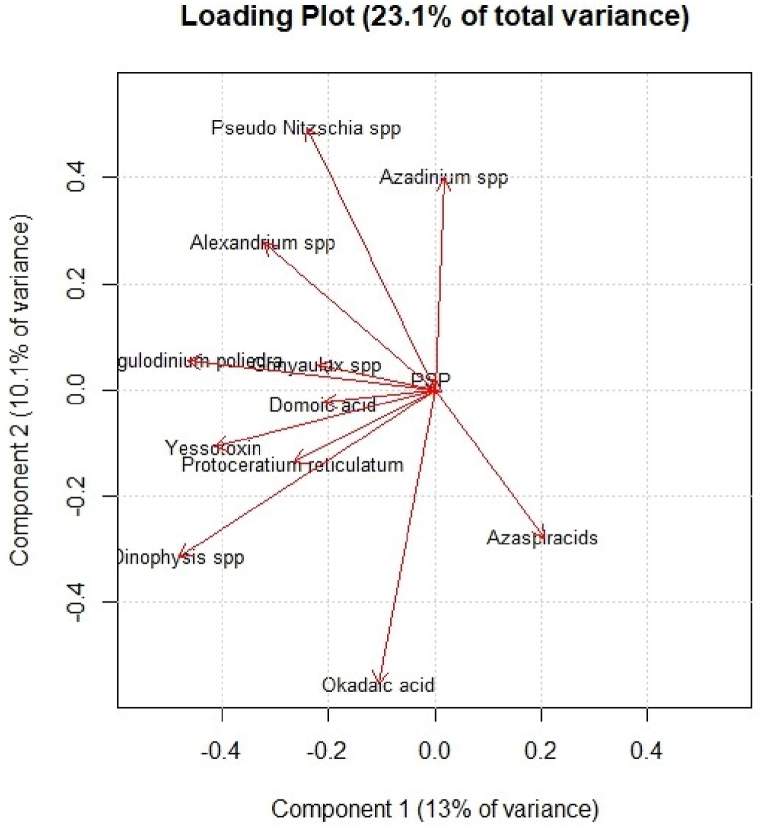
Loading plot in the plane of the two lowest-order PCs. Original variables of algae and biotoxins are shown.

A positive correlation between algae and their biotoxins is highlighted in the loading plot shown in Fig. 6: most of the variables are located towards negative values of PC1, but the correlation is stronger in the case of yessotoxins and their causative algae *Gonyaulax* spp*., Lingulodinium polyedrum* and *Protoceratium reticulatum*, as well as okadaic acid and algae of genus *Dinophysis*. Since PC1 and PC2 explain a reduced percentage of variance (because of the limited correlation structure characterising original variables), it is important to remember that these graphics show only a small fraction of the total information, thus the conclusions we may draw are attributable only to the part of information shown in these graphics.

**Fig. 7:**
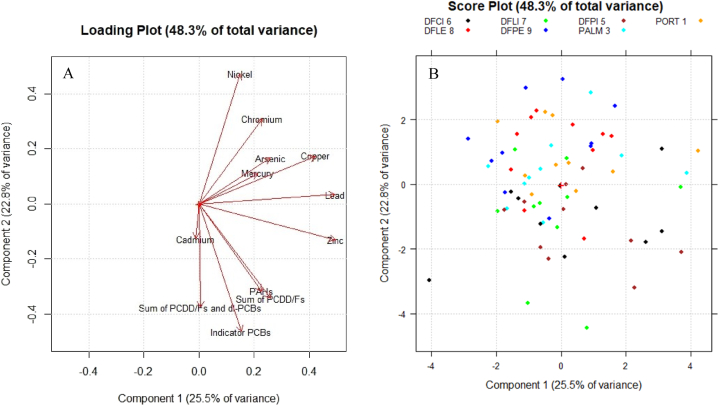
(A) Loading plot in the plane of the two lowest-order PCs. Original variables of chemical pollutants are shown. (B) Score plot in the plane of the two lowest-order PCs. Samples are identified with different colours on the basis of the monitoring station of origin. (For interpretation of the references to colour in this figure legend, the reader is referred to the Web version of this article.)

Further PCA were carried out on chemical variables. Fig. 7A shows the loading plot in the plane of the two lowest-order PCs. It can be noticed that all the variables are located towards positive values of PC1: this component distinguishes samples as a function of their global content of contaminants. Instead, PC2 differentiates contaminants of inorganic origin, located mainly along the positive semiaxis, from those of organic origin, located along the negative semiaxis.

Unlike the outcomes from biotoxicological and microbiological variables, in the score plot in Fig. 7B, it was possible to detect a clear differentiation between samples collected from different monitoring stations, based on chemical variables. Samples coming from sites DFPI 5, DFCI 6 and DFLI 7, located in the inner part of the outer dam, are mostly situated towards negative values of PC2, whereas the ones coming from PORT 1, PALM 3, DFLE 8 and DFPE 9, located in the outer part of the Gulf of La Spezia, are situated towards positive values of the same component. Comparing this graphic with the loading plot in Fig. 7A, it is possible to deduce that the first sites are characterised by a greater amount of dioxins, PCBs and PAHs; instead, heavy metals are more common in the latter sites. The entire bay of La Spezia is, in fact, strongly anthropized. There are multiple activities linked to water pollution, such as commercial and tourist ports, military arsenal, areas for shipbuilding, as well as industrial and urban effluents. In all of this, sea currents probably have an important role in the distribution of contaminants: two factors combined (the wind and the thermal gradient produced by the electric power plant) are responsible for water renewal, from the inner parts of the gulf to the outer dam, whereas other currents promote the mixing of the water inside the outer dam with the open sea [81].

The numerous analyses carried out by the competent authority make it possible to monitor the situation in the Gulf over time and protect the consumer from any risks associated with the consumption of bivalve molluscs from the Ligurian Sea.

Unfortunately, our elaboration does not allow to highlight a temporal link between the presence of algae in the waters and the relative accumulation in the molluscs. However, it does suggest the possibility of reducing the sampling points and the frequency as regards the microbiological and biotoxicological analyses during the winter season, while it is advisable maintain the current state of controls on environmental contaminants.

## 5 Conclusions

Between 2015 and 2021, a total of 13105 analyses were made on 798 samples.

Analyses of the main components conducted on the 31 variables measured for 6 years on samples of *Mytilus galloprovincialis* highlighted some interesting aspects. First, seasonal trends related to the development of algae and the presence of the related biotoxins were highlighted: greater blooms were found when temperatures were milder, especially during the spring months, except for *Azadinium* spp. algae that are known to be located in seas with colder temperatures [2]. This could lead to a reduction in the frequency of biotoxicological analyses during the winter season. Since there were no significant differences between the monitoring stations, also a reduction in sampling points can be justified.

The opposite case was represented by the results obtained from the chemical pollutant data processing, which showed distinctions between the sampling sites, linked to the nature – organic or inorganic – of the most common pollutants. In the future, it would be interesting to study the reasons for this differentiation, considering the proximity of the stations to the port of La Spezia and the industrial areas, as well as the routes of the ships.

Excluding the year 2017, which most distinguished itself from the others due to atypical meteorological conditions, the situation, regarding the algae and biotoxins examined, was almost constant over time. This could steer to focus, in the future, on the study of emerging toxins in order to investigate their distribution profile and behaviour over time. A further possible development of the present work, which was not investigated in the present study due to low sampling frequency, could be to study alga-biotoxin correlations considering a time shift between the detection of the alga and the presence of the toxin.

It is also important to remark that the statistical analysis was carried out on a dataset that covers a limited period of time (6 years): to confirm the correlations highlighted and to establish any causal links, it will be necessary to continue this study on a longer period of time, as well as pursuing mechanistic investigations of chemical and biological nature.

## Author contribution statement

Valentina Ciccotelli; Erica Vaccaro: Conceived and designed the experiments; Performed the experiments; Analysed and interpreted the data; Contributed reagents, materials, analysis tools or data; Wrote the paper.

Paolo Oliveri: Conceived and designed the experiments; Analysed and interpreted the data; Contributed reagents, materials, analysis tools or data; Wrote the paper.

Roberta Battistini; Cristina Capelli: Conceived and designed the experiments; Performed the experiments; Contributed reagents, materials, analysis tools or data; Wrote the paper.

Stefano Lottici; Nunzia Melchiorre; Elena Smirnova; Marta Ferro; Chiara Masotti: Conceived and designed the experiments; Performed the experiments; Contributed reagents, materials, analysis tools or data.

Erica Costa; Barbara Betti: Conceived and designed the experiments; Analysed and interpreted the data; Contributed reagents, materials, analysis tools or data.

Barbara Vivaldi; Laura Serracca; Francesco Iacona; Mino Orlandi; Carlo Ercolini: Conceived and designed the experiments; Contributed reagents, materials, analysis tools or data.

## Data availability statement

Data included in article/supplementary material/referenced in article.

## Additional information

Supplementary content related to this article has been published online at [URL].

## Declaration of competing interest

The authors declare the following financial interests/personal relationships which may be considered as potential competing interests:Valentina Ciccotelli reports article publishing charges was provided by Zooprophylactic Institute of Piemonte Liguria and Valle d’Aosta.
