# Supplementary material for: Shellfish sanitation monitoring in La Spezia gulf: Chemometric evaluation of data from 2015 to 2021
Source: Heliyon. 2023 Jun 15;9(6):e17032. doi: 10.1016/j.heliyon.2023.e17032 (PMC10293677; doi:10.1016/j.heliyon.2023.e17032)
Supplement: Multimedia component 1 [file mmc1.docx]

**Supplementary material**

**Algae temporal trend**

**Fig. S1.** Temporal trend of *Alexandrium* spp. abundances in seawater collected from seven monitoring stations in the Gulf of La Spezia (PORT 1, PALM 3, DFPI 5, DFCI 6, DFLI 7, DFLE 8, DFPE 9) for 6 years (January 2015 – December 2020). All data are reported in logarithmic scale (log cell L^-1^). Limit of quantification (LOQ) is set at 20 cell L^-1^ and attention level at 500 cell L^-1^.

**Fig. S2**. Temporal trend of *Pseudo-nitzschia* spp. abundances in seawater collected from seven monitoring stations in the Gulf of La Spezia (PORT 1, PALM 3, DFPI 5, DFCI 6, DFLI 7, DFLE 8, DFPE 9) for 6 years (January 2015 – December 2020). All data are reported in logarithmic scale (log cell L^-1^). Limit of quantification (LOQ) is set at 20 cell L^-1^ and attention level at 500,000 cell L^-1^.

**Fig. S3.** Temporal trend of *Dinophysis* spp. abundances in seawater collected from seven monitoring stations in the Gulf of La Spezia (PORT 1, PALM 3, DFPI 5, DFCI 6, DFLI 7, DFLE 8, DFPE 9) for 6 years (January 2015 – December 2020). All data are reported in logarithmic scale (log cell L^-1^). Limit of quantification (LOQ) is set at 20 cell L^-1^ and attention level at 500 cell L^-1^.

**Fig. S4.** Temporal trend of *Gonyaulax* spp. abundances in seawater collected from seven monitoring stations in the Gulf of La Spezia (PORT 1, PALM 3, DFPI 5, DFCI 6, DFLI 7, DFLE 8, DFPE 9) for 6 years (January 2015 – December 2020). All data are reported in logarithmic scale (log cell L^-1^). Limit of quantification (LOQ) is set at 20 cell L^-1^ and attention level at 500 cell L^-1^.

**Fig. S5.** Temporal trend of *L. polyedra* abundances in seawater collected from seven monitoring stations in the Gulf of La Spezia (PORT 1, PALM 3, DFPI 5, DFCI 6, DFLI 7, DFLE 8, DFPE 9) for 6 years (January 2015 – December 2020). All data are reported in logarithmic scale (log cell L^-1^). Limit of quantification (LOQ) is set at 20 cell L^-1^ and attention level at 500 cell L^-1^.

**Fig. S6.** Temporal trend of *P. reticulatum* abundances in seawater collected from seven monitoring stations in the Gulf of La Spezia (PORT 1, PALM 3, DFPI 5, DFCI 6, DFLI 7, DFLE 8, DFPE 9) for 6 years (January 2015 – December 2020). All data are reported in logarithmic scale (log cell L^-1^). Limit of quantification (LOQ) is set at 20 cell L^-1^ and attention level at 500 cell L^-1^.

**Fig. S7.** Temporal trend of *Azadinium* spp. abundances in seawater collected from seven monitoring stations in the Gulf of La Spezia (PORT 1, PALM 3, DFPI 5, DFCI 6, DFLI 7, DFLE 8, DFPE 9) for 6 years (January 2015 – December 2020). All data are reported in logarithmic scale (log cell L^-1^). Limit of quantification (LOQ) is set at 20 cell L^-1^ and attention level at 10,000 cell L^-1^.

**Biotoxins temporal trend**

A

B

**Fig. S8.** Temporal trend of (A) Domoic acid, (B) Okadaic acid and (C) Yessotoxins in mussels collected from seven monitoring stations in the Gulf of La Spezia (PORT 1, PALM 3, DFPI 5, DFCI 6, DFLI 7, DFLE 8, DFPE 9) for 6 and a half years (January 2015 – June 2021). Limit of quantification (LOQ) is set at 5 mg kg^-1^ for DA, 20 µg OA equivalents kg^-1^ for OA and 0.05 mg YTX equivalents kg^-1^ for YTX. Azaspiracid concentration was always below the detection limit.

**Temporal trend of microbiological parameters**

**Fig. S9.** Temporal trend of *E. coli* in mussels collected from seven monitoring stations in the Gulf of La Spezia (PORT1, PALM 3, DFPI 5, DFCI 6, DFLI 7, DFLE 8, DFPE 9) for 6 and a half years (January 2015 – June 2021). All data are reported in logarithmic scale (log MPN/100 g). Limit of quantification (LOQ) is set at 18 MPN/100 g.

**Fig. S10.** Temporal trend of (A) norovirus and (B) *Salmonella* spp. in mussels collected from seven monitoring stations in the Gulf of La Spezia (PORT1, PALM 3, DFPI 5, DFCI 6, DFLI 7, DFLE 8, DFPE 9) for 6 and a half years (January 2015 – June 2021). All data are expressed as presence/absence: positive samples are shown with points above 0, whereas negative samples are below 0. *V. parahaemolyticus, V. cholerae, V. vulnificus* and HAV were always negative.

**Temporal trend of chemical parameters**

**Fig. S11.** Temporal trend of (A) Hg, (B) Cd, (C) Pb, (D) As, (E) Zn, (F) Cu, (G) Ni and (H) Cr in mussels collected from seven monitoring stations in the Gulf of La Spezia (PORT1, PALM 3, DFPI 5, DFCI 6, DFLI 7, DFLE 8, DFPE 9) for 6 years (May 2015 – June 2020).

**Fig. S12**. Temporal trend of (A) PCDD/Fs, (B) indicator PCBs and (C) sum of PCDD/Fs and dl-PCBs in mussels collected from seven monitoring stations in the Gulf of La Spezia (PORT1, PALM 3, DFPI 5, DFCI 6, DFLI 7, DFLE 8, DFPE 9) for 6 years (May 2015 – June 2020).

**Fig. S13.** Temporal trend of polycyclic aromatic hydrocarbons (PAHs) in mussels collected from seven monitoring stations in the Gulf of La Spezia (PORT1, PALM 3, DFPI 5, DFCI 6, DFLI 7, DFLE 8, DFPE 9) for 6 years (May 2015 – June 2020).
